# N-acetyl cysteine, inulin and the two as a combined therapy ameliorate cognitive decline in testosterone-deprived rats

**DOI:** 10.18632/aging.101989

**Published:** 2019-06-03

**Authors:** Titikorn Chunchai, Puntarik Keawtep, Apiwan Arinno, Napatsorn Saiyasit, Dillon Prus, Nattayaporn Apaijai, Wasana Pratchayasakul, Nipon Chattipakorn, Siriporn C. Chattipakorn

**Affiliations:** 1 Neurophysiology Unit, Cardiac Electrophysiology Research and Training Center, Faculty of Medicine, Chiang Mai University, Chiang Mai 50200, Thailand; 2 Cardiac Electrophysiology Unit, Department of Physiology Faculty of Medicine, Chiang Mai University, Chiang Mai 50200, Thailand; 3 Department of Oral Biology and Diagnostic Sciences, Faculty of Dentistry, Chiang Mai University, Chiang Mai 50200, Thailand

**Keywords:** cognition, testosterone deprivation, gut dysbiosis, glia, apoptosis, hippocampal plasticity

## Abstract

Our previous studies reported that testosterone-deprived rats developed cognitive decline as a result of increased brain oxidative stress, microglia hyperactivity, and hippocampal dysplasticity. In addition, gut dysbiosis occurred in these rats. Previous studies demonstrated that n-acetyl cysteine (NAC) and a prebiotic (inulin) improved cognition in several pathological conditions. However, its effects on cognition in the testosterone-deprived condition have never been investigated. This study hypothesized that the administration of NAC, inulin, and a combined therapy improved cognition in castrated rats. Here we report that metabolic disturbance was not observed in the ORX rats, but gut dysbiosis was found in these rats. ORX rats developed blood-brain-barrier (BBB) breakdown, and increased brain oxidative stress as indicated by increased hippocampal production of reactive oxygen species (ROS) and an increase in brain malondialdehyde level. ORX rats also demonstrated glia hyperactivation, resulting in hippocampal apoptosis, hippocampal dysplasticity, and cognitive decline. All treatments equally ameliorated cognitive decline by improving gut dysbiosis, alleviating BBB dysfunction, decreasing hippocampal ROS production, decreasing hippocampal apoptosis, and reducing microglia and astrocyte activity. These findings suggest that NAC, inulin, and the combined therapy ameliorated the deleterious effects on the brain in castrated male rats similar to those treated with testosterone.

## Introduction

Testosterone, a steroid sex hormone, plays an important role in cognitive function by modulating the number of dendritic spines, and mediating the release of neurotransmitters [1], as well as being involved in synaptic formation in the brain [2]. Testosterone deficiency has been an independent risk factor for cognitive decline [3]. Male rodents with an orchiectomy demonstrated dendritic spine loss [4], which could be restored by testosterone or Dihydrotestosterone (DHT) replacement [4,5]. Similar to those animal studies, previous studies in men demonstrated that low testosterone levels have a positive correlation with a decline in learning and memory [6]. Furthermore, testosterone replacement could improve performance in a verbal memory test and spatial learning and memory in healthy hypogonadal men or men with Alzheimer’s disease [7].

In addition to testosterone, gut microbiota, a group of beneficial microbes living inside the gastrointestinal tract, have been shown to play an important role in cognitive function in obesity, Parkinson’s disease, Alzheimer’s disease, traumatic brain injury, and inflammatory bowel disease [8]. The imbalance of gut microbiota or called “gut dysbiosis” in these conditions were indicated by an increase in the ratio of *Firmicutes* to *Bacteroidetes* (F/B ratio) and *Proteobacteria* [9,10]. This gut dysbiosis has been associated with cognitive dysfunction [11]. The modulation of gut microbiota by prebiotics could be an effective therapeutic strategy to improve the cognitive impairment [12]. The beneficial effects of prebiotics were also found to improve metabolic disturbance in obese animals by altering the composition and metabolism of gut microbiota [13].

The functional foods which include the fiber-containing foods such as inulin, have the ability to improve health and wellbeing [14]. Inulin acts as a prebiotic by being resistant to gastric acidity and enzymes, and being undigested, but it can undergo fermentation by bacteria in the colon. It has been shown that inulin supplement increased the growth of *Bifidobacterium* [15] and *Lactobacillus* species [16], leading to the increased of short-chain fatty acids (SCFAs) [17,18]. The diet of treated rats was prepared by adding 10% of inulin fiber into the control diet (by weight). Previous study reported that consumption of 10% inulin for 4 weeks markedly shifted the composition of gut microbiota and improved intestinal function [19]. Several studies also showed the beneficial effects of inulin on cognitive function in both animals and humans [20,21]. However, the controversial results reported that prebiotic administration altered blood parameters, but no benefit was observed in cognitive behavior or sleep quality [22].

Microglia, the brain resident macrophages, play a crucial role in neurodegenerative disorders. For example: 1) Microglia excessively prune synapses and increase pro-inflammatory cytokines in models of Alzheimer's disease [23,24]. 2) Microglial hyperactivity has been associated with cognitive decline in obesity [25]. Interestingly, recent studies illustrated a communication link between microglial function and host microbiota [26,27]. In addition, reactive oxygen species (ROS) and oxidative stress levels is known to increase in testosterone-deprived condition [28,29]. The oxidative stress can induce microglia activation [30] and inhibit long-term potentiation (LTP), resulting to cognitive impairment [31]. Several studies have demonstrated the protective effects of N-acetyl cysteine (NAC) on microglial activation and inflammation in both *in vitro* and *in vivo* studies [32–35]. Gunther and colleagues showed that NAC decreased neuronal degeneration, decreased neuronal apoptosis, and increased anti-oxidative enzymes [35]. Previous study also demonstrated that macrophage/microglia activation was associated with an increase in circulating oxidative stress and inflammation in type 2 diabetes mellitus (T2DM) and this activation was minimized by NAC [32].

Despite these previous findings, the effects of NAC, inulin, and the two as a combined therapy on cognitive function, gut microbiota alteration, peripheral insulin sensitivity, hippocampal synaptic plasticity, hippocampal oxidative stress, microglial/astrocyte morphology, and hippocampal apoptosis in castrated male rats has never been investigated. Therefore, this study aimed to investigate the effects of testosterone replacement, NAC, inulin and the combined therapy on cognitive function and brain pathology in castrated male rats.

## RESULTS

### NAC, inulin, and the combined therapy improved gut dysbiosis in ORX rats

**Figure 1:**
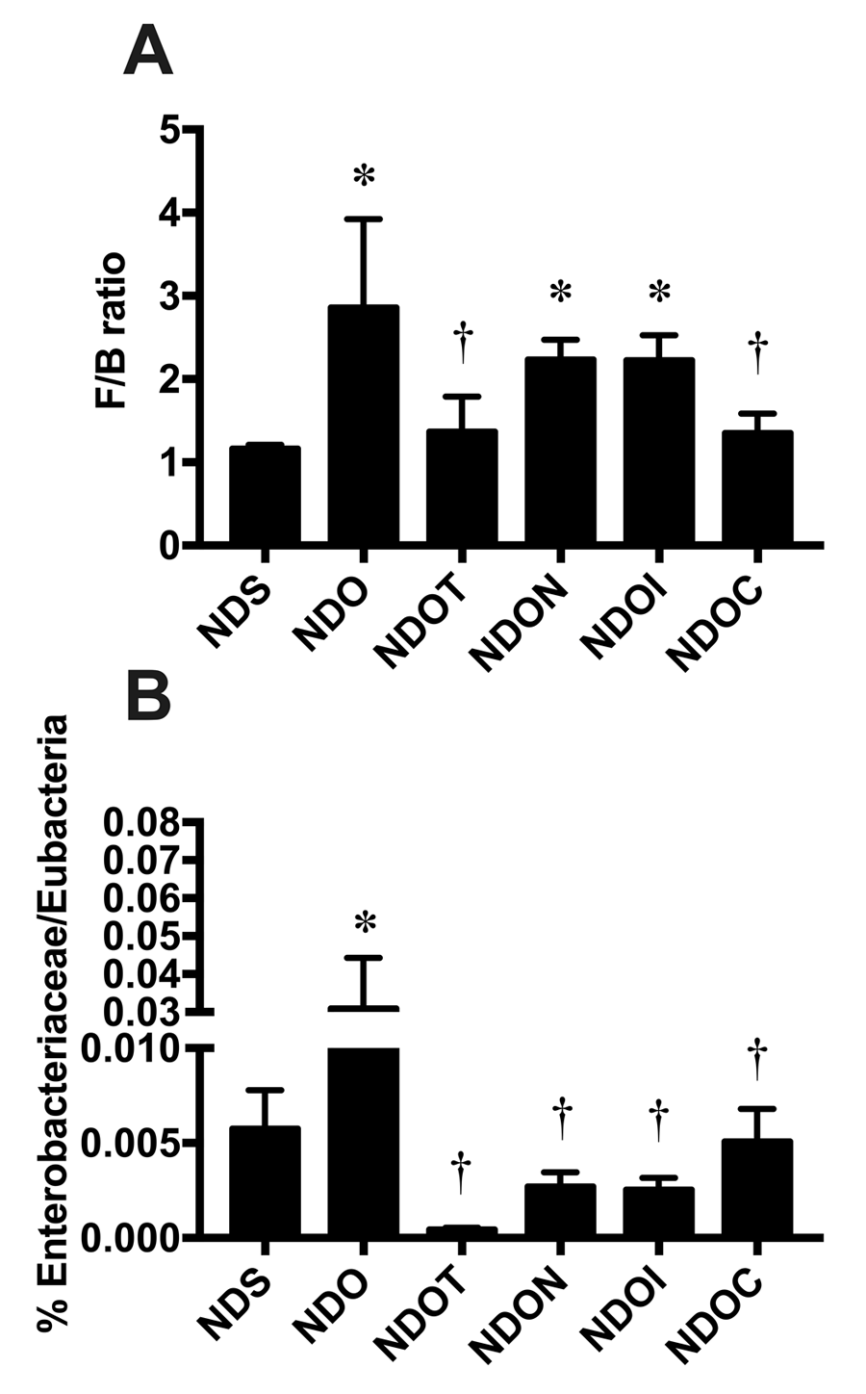
**The effects of NAC, inulin and combined therapy on gut dysbiosis in rats with testosterone deprivation.** (**A**) F/B ratio and (**B**) Enterobacteriaceae were normalized by Eubecteria. NDS: rats with sham operation; NDO: rats with orchiectomy; NDOT: rats with orchiectomy receiving testosterone replacement; NDON: rats with orchiectomy receiving NAC treatment; NDOI: rats with orchiectomy receiving inulin treatment; NDOC: rats with orchiectomy receiving the combined therapy (the combination of NAC and inulin) (N=6 in each group) *p<0.05 in comparison with the NDS, †p<0.05 in comparison with the NDS.

ORX rats had significantly decreased testosterone levels when compared to that of sham-operated rats (Table 1). These findings indicated that a bilateral orchiectomy is an effective model for testosterone deprivation. To determine the effects of NAC, inulin, and the combined therapy on alteration in gut microbiota in ORX rats, the bacterial microbiota was measured from fecal pellets. The results demonstrated that ORX rats treated with vehicle had gut dysbiosis, as indicated by an increased F/B ratio, when compared to sham-operated rats (Figure 1A). F/B ratio of ORX rats treated with testosterone replacement and the combined therapy, but not NAC or inulin alone, were significantly reduced, when compared to that of ORX rats treated with vehicle (Figure 1A). Similar to the F/B ratio, *Enterobacteriaceae* levels of ORX rats treated with the vehicle significantly increased. That increase was significantly ameliorated by all treatments (Figure 1B). These findings suggested that testosterone-deprived rats developed gut dysbiosis. NAC, and inulin attenuated gut dysbiosis, but the combined therapy and testosterone replacement had better efficacy to restore the balance of gut microbiota in the testosterone-deprived condition.

**Table 1:** The metabolic parameters of sham-operated rats and ORX rats with either testosterone replacement, NAC, inulin or combined therapy.

**Metabolic parameters**		**NDS**		**NDO**		**NDOT**		**NDON**		**NDOI**		**NDOC**
Body weight (g)		566±23		464±22*		550±24†		483±26*		470±10*		491±9*
Food intake (g/day)		18.9±0.4		16.3±0.4*		17.5±0.9		16.8±0.1*		14.9±0.4*†		16.1±0.2*
Visceral fat (g)		35±2		22±2*		24±3		19±3*		17±3*†		21±3*
Seminal vesicle (g)		1.35±0.12		0.12±0.01*		1.84±0.14		0.07±0.02*		0.13±0.01*		0.20±0.02*
Testosterone (pg/ml)		2.28±0.3		0.02±0.01*		2.27±0.1		0.02±0.01*		0.02±0.01*		0.02±0.01*
Plasma glucose (mg/dl)		132±4		142±5		136±9		124±4		127±7		127±5
Plasma insulin (ng/ml)		4.8±0.8		5.1±1		5.2±0.8		4.5±1		4.3±0.5		4.5±0.5
HOMA index		42.3±10		51.0±14		53.8±11		49.9±12		54.7±10		53.6±6
Plasma glucose AUC (AUCg) (mg/dl×min×10^4^)		2.3±0.1		2.3±0.1		2.3±0.1		2.1±0.2		2.1±0.1		2.3±0.1
Plasma total cholesterol (mg/dl)		73±4		79±3		80±8		87±8		86±3		89±4
Plasma total triglyceride (mg/dl)		73±4		78±4		63±6		39±15*†		21±2*†		36±5*†
Plasma LDL cholesterol (mg/dl)		58.7±5		52.3±2		50.9±6		47.8±6		54.6±10		55.2±4
Plasma HDL cholesterol (mg/dl)		44.2±5		39.3±2		43.5±6		41.6±6		39.8±1		39.1±4

NDS: rats with sham operation; NDO: rats with orchiectomy; NDOT: rats with orchiectomy receiving testosterone replacement; NDON: rats with orchiectomy receiving NAC treatment; NDOI: rats with orchiectomy receiving inulin treatment; NDOC: rats with orchiectomy receiving the combined therapy (the combination of NAC and inulin) (N=6 of each group) *P<0.05 in comparison with the NDS group; †*P*<0.05 in comparison with the NDO group

### The effects of NAC, inulin and the combined therapy on metabolic profiles in ORX rats

ORX rats showed significantly decreased body weight, food intake, and visceral fat weight when compared to those of sham-operated rats (Table 1). To investigate the effects of testosterone deprivation on peripheral insulin sensitivity, plasma glucose levels, plasma insulin levels, HOMA-IR index and the area under the curve of OGTT were measured. We found that the plasma glucose levels, plasma insulin levels, HOMA-IR index and the area under the curve of OGTT were not different among all groups (Table 1). These findings suggested that testosterone deprivation did not impair peripheral insulin sensitivity.

Similar to insulin sensitivity, plasma triglyceride, plasma cholesterol, plasma HDL and LDL levels were not significant difference between ORX rats and sham-operated rats. However, treatment with NAC, inulin and combined therapy, but not testosterone, in ORX rats significantly decreased the plasma triglyceride levels, when compared to sham-operated rats and ORX rats treated with vehicle (Table 1). These findings indicated that NAC, inulin, and combined therapy decreased plasma triglyceride levels in testosterone-deprived rats.

### NAC, inulin, and the combined therapy improved blood brain barrier integrity, hippocampal oxidative stress, and hippocampal inflammation in ORX rats

**Figure 2:**
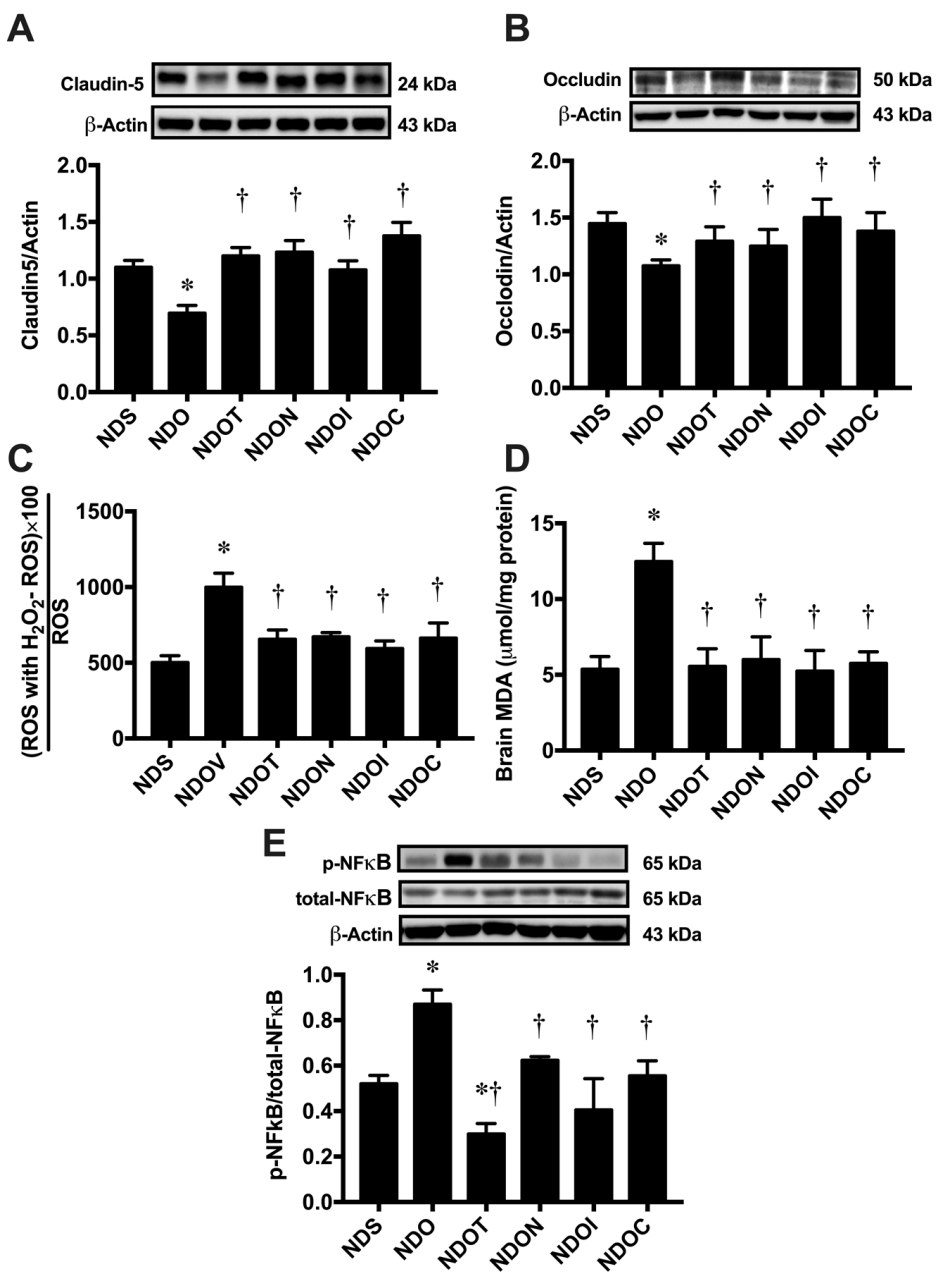
**The effects of NAC, inulin and combined therapy on blood-brain barrier integrity, hippocampal oxidative stress and hippocampal inflammation in rats with Testosterone deprivation.** (**A**) *Upper panels*: the representative bands of claudin-5 blotting. *Lower panel*: The expression of the hippocampal claudin-5 protein. (**B**) *Upper panels*: the representative bands of occludin blotting. *Lower panel*: The expression of hippocampal occludin protein. (**C**) Hippocampal oxidative stress as indicated by hippocampal ROS level. (**D**) Brain oxidative stress as indicated by brain MDA level. (**E**) *Upper panels*: the representative bands of p-NFĸb, total-NFĸB and actin. *Lower panel*: The expression of p-NFĸb per total-NFĸB ratio. NDS: rats with sham operation; NDO: rats with orchiectomy; NDOT: rats with orchiectomy receiving testosterone replacement; NDON: rats with orchiectomy receiving NAC treatment; NDOI: rats with orchiectomy receiving inulin treatment; NDOC: rats with orchiectomy receiving a combined therapy (the combination of NAC and inulin) (N=6 of each group) *p<0.05 in comparison with the NDS, †p<0.05 in comparison with the NDS.

To investigate blood-brain-barrier permeabilization, the expression of hippocampal tight junction proteins including claudin-5 and occludin were measured by immunoblotting. We found that the expression of both hippocampal claudin-5 and occludin significantly decreased in ORX rats, when compared with sham-operated rats (Figure 2A and B). Testosterone replacement, and treatment with NAC, inulin, and the combined therapy in ORX rats led to equally increase claudin-5 and occludin expression (Figure 2A and B), suggesting that testosterone replacement, NAC, inulin, and the combined therapy improved BBB integrity in testosterone-deprived condition.

Regarding to level of oxidative stress, hippocampal ROS production and brain MDA levels showed a significant increase in ORX rats, when compared to sham-operated rats (Figure 2C and D). These parameters were restored to within normal limits by testosterone replacement, NAC, inulin, and the combined therapy (Figure 2C and D).

Next, we determined the level of hippocampal inflammation by immunoblotting. We found that the p-NFĸb/total-NFĸB ratio significantly increased in ORX rats when compared to sham-operated rats (Figure 2E). All treatments equally decreased p-NFĸb/ total-NFĸB ratio in ORX rats (Figure 2E). These findings suggested that hippocampal and brain oxidative stress as well as brain inflammation occurred in the testosterone-deprived condition, which were ameliorated by testosterone, NAC, inulin and the combined therapy.

### NAC, inulin and the combined therapy improved glial morphological changes in ORX rats

**Figure 3:**
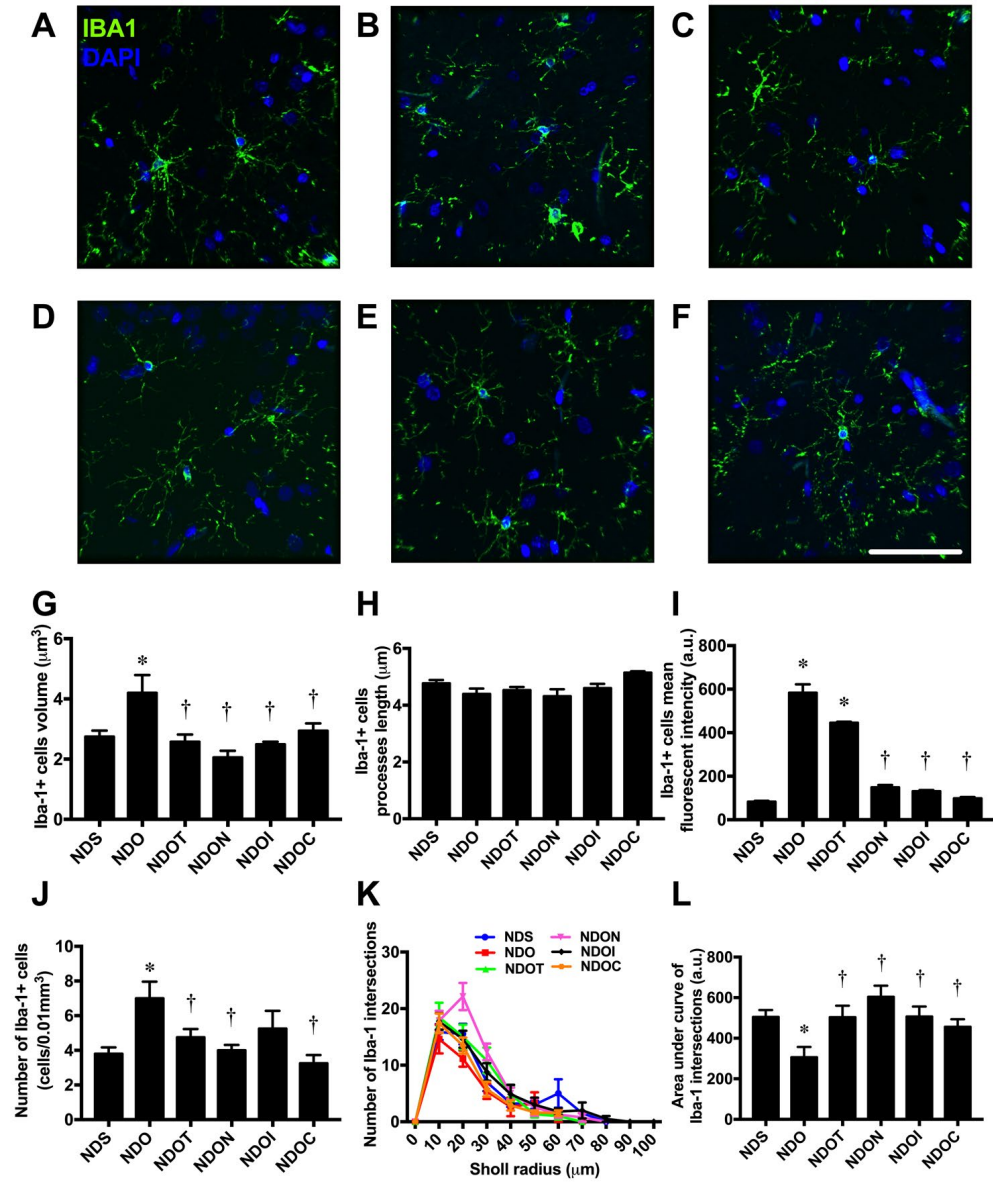
**The effects of NAC, inulin and combined therapy on microglial morphology in rats with testosterone deprivation.** (**A**-**F**) Representative images of Iba-1 and DAPI immunofluorescence under confocal microscopy at CA1 of the hippocampus of NDS, NDO, NDOT, NDON, NDOI, and NDOC respectively (bar = 50 𝜇m). (**G**) Size of microglial cells as indicated by Iba-1 positive cell volume. (**H**) Length of Microglial processes as indicating Iba1 positive cells processes length. (**I**) Mean fluorescent intensity of Iba-1 positive cells. (**J**) Number of microglial cell as indicated by number of Iba-1 positive cells. (**K**-**L**) The ramification the microglial cells as indicated by Sholl analysis and area under the curve of Iba-1 intersection respectively. NDS: rats with sham operation; NDO: rats with orchiectomy; NDOT: rats with orchiectomy receiving testosterone replacement; NDON: rats with orchiectomy receiving NAC treatment; NDOI: rats with orchiectomy receiving inulin treatment; NDOC: rats with orchiectomy receiving the combined therapy (the combination of NAC and inulin) (N=6 in each group) *p<0.05 in comparison with the NDS, †p<0.05 in comparison with the NDS.

To determine the morphology of the microglia at the CA1 region of the hippocampus, brain sections were immunostained with anti-Iba1 and DAPI (Figure 3A-F). The results demonstrated that the cell volume of Iba1 positive cells significantly increased in ORX rats, when compared with sham-operated rats (Figure 3G). These changes were ameliorated by all treatments. However, the lengths of Iba1 positive cell processes did not differ between all groups (Figure 3H).

ORX rats also exhibited significantly increased numbers of Iba1 positive cells and the mean fluorescent intensity of Iba1 positive cells, when compared with sham-operated rats (Figure 3I-J). The complexity of microglial morphology was further investigated by Sholl analysis. The microglial ramification of ORX rats was decreased as indicated by the decline in number of Iba1 intersections and area under the curve of Iba1 intersections. All treatments equally increased microglial ramifications (Figures 3K and L).

**Figure 4:**
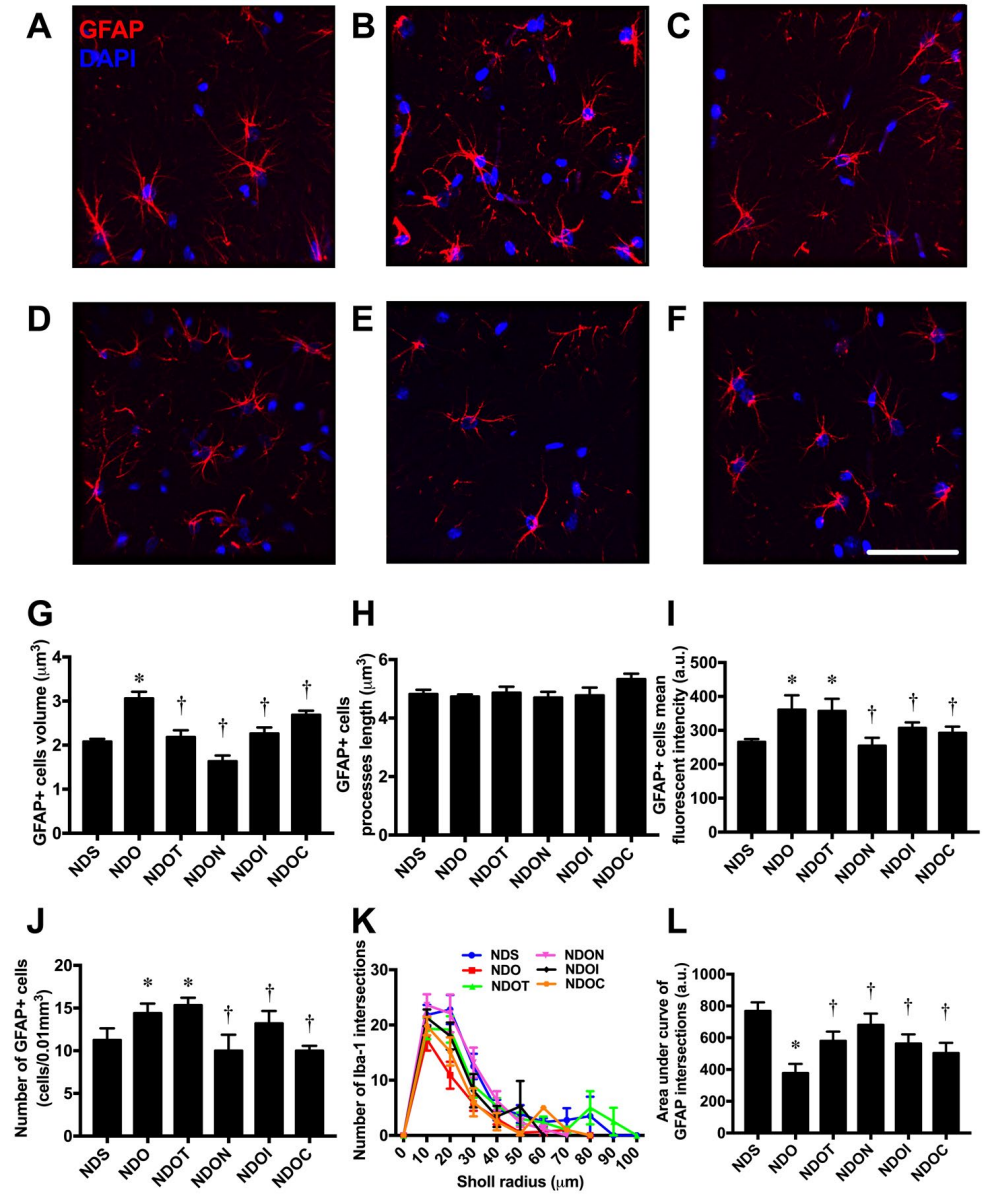
**The effects of NAC, inulin and combined therapy on astrocyte morphology in rats with testosterone deprivation.** (**A**-**F**) Representative images of GFAP and DAPI immunofluorescence under confocal microscopy at CA1 of the hippocampus of NDS, NDO, NDOT, NDON, NDOI, and NDOC respectively (bar = 50 𝜇m). (**G**) Size of astrocyte cells as indicated by GFAP positive cell volume. (**H**) Length of astrocyte processes as indicated by GFAP positive cells processes length. (**I)** Mean fluorescent intensity of GFAP positive cells. (**J**) Number of astrocyte cells as indicated by number of GFAP positive cells. (**K**-**L**) The ramification of astrocyte cells as indicated by Sholl analysis and area under the curve of GFAP intersection respectively. NDS: rats with sham operation; NDO: rats with orchiectomy; NDOT: rats with orchiectomy receiving testosterone replacement; NDON: rats with orchiectomy receiving NAC treatment; NDOI: rats with orchiectomy receiving inulin treatment; NDOC: rats with orchiectomy receiving the combined therapy (the combination of NAC and inulin) (N=6 of each group) *p<0.05 in comparison with the NDS, †p<0.05 in comparison with the NDS.

In addition to microglia, we investigated astrocyte morphology. The morphology of the astrocytes was shown by immunostaining of anti-GFAP and DAPI (Figure 4A-F). An increase in cell volume of GFAP positive cells in ORX rats was observed in ORX rats, when compared with sham-operated rats. All treatments decreased cell size of GFAP positive cells in ORX rats (Figure 4G). Although we found that the lengths of GFAP positive cells were not significantly different among all groups (Figure 4H), the mean fluorescence intensity of GFAP positive cells significantly increased in both ORX-fed rats treated with vehicle and testosterone. NAC, inulin, and the combined therapy reduced the mean fluorescence intensity of GFAP positive cells in ORX rats (Figure 4I). Similar to the mean fluorescent intensity, ORX-fed rats treated with either the vehicle or testosterone had a significantly increased number of GFAP positive cells. This increment was attenuated by treatment with NAC, inulin, or the combined therapy (Figure 4J). The complexity of astrocyte morphology was further investigated by Sholl analysis. The ramification of the astrocytes from ORX rats decreased, as indicated by the decline in number of GFAP intersections and area under the curve of GFAP intersections. All treatments led to an equal increase in astrocyte ramification (Figures 4K and L). All of these findings demonstrated that testosterone deprivation altered microglial and astrocyte morphology, and these changes were attenuated by all treatments.

### NAC, inulin, and the combined therapy improved hippocampal apoptosis in ORX rats

**Figure 5:**
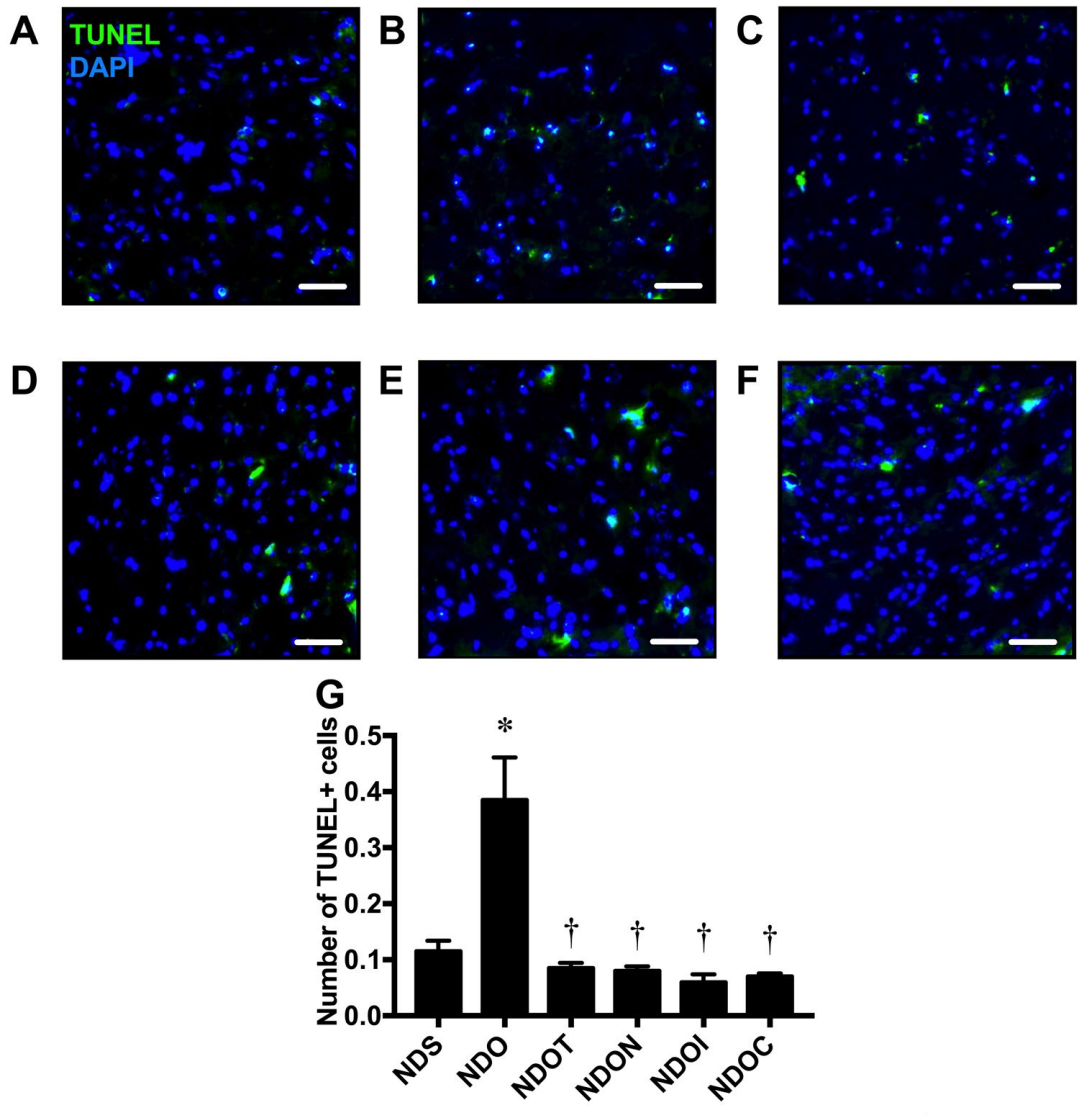
**The effects of NAC, inulin and the combined therapy on hippocampal apoptosis in rats with testosterone deprivation.** (**A**-**F**) Representative images of TUNEL positive cells and DAPI immunofluorescence under confocal microscopy at CA1 of the hippocampus of NDS, NDO, NDOT, NDON, NDOI, and NDOC respectively (scale bar = 50 µm). (**G**) Hippocampal apoptosis as indicated by the co-localization of the number of hippocampal TUNEL positive cells and DAPI. NDS: rats with sham operation; NDO: rats with orchiectomy; NDOT: rats with orchiectomy receiving testosterone replacement; NDON: rats with orchiectomy receiving NAC treatment; NDOI: rats with orchiectomy receiving inulin treatment; NDOC: rats with orchiectomy receiving the combined therapy (the combination of NAC and inulin) (N=6 of each group) *p<0.05 in comparison with the NDS, †p<0.05 in comparison with the NDS.

To determine hippocampal apoptosis, brain sections were further investigated using the TACS 2TdT-Fluor *in situ* apoptosis detection kit. Colocalization of TUNEL positive cells and DAPI was used to identify apoptosis in the hippocampi (Figure 5A-F). We found that ORX rats had significantly increased numbers of apoptotic cells as indicated by the increased number of TUNEL positive cells (Figure 5B and G). Testosterone replacement, NAC, inulin, or the combined therapy equally decreased TUNEL positive cells in ORX rats (Figure 5C-G), suggesting testosterone replacement, NAC, inulin or the combination of both NAC and inulin attenuated hippocampal apoptosis in testosterone-deprived rats.

### NAC, inulin and the combined therapy improved hippocampal dysplasticity leading to the improvement of cognition in ORX rats.

**Figure 6:**
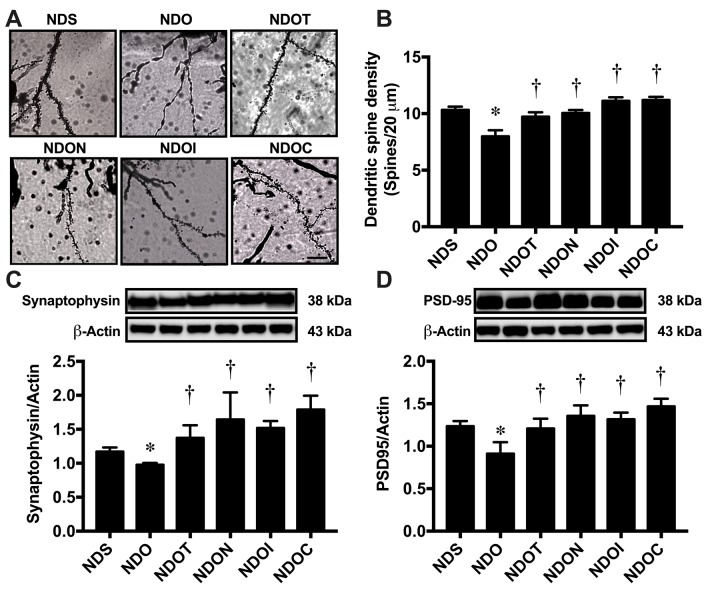
**The effects of NAC, inulin and combined therapy on hippocampal plasticity in rats with testosterone deprivation.** (**A**) Representative images of dendritic spines under microscopy at CA1 of the hippocampus of NDS, NDO, NDOT, NDON, NDOI, and NDOC respectively (scale bar = 20 µm). (**B**) Mean dendritic spine density. (**C**) *Upper pane*: representative immunoblotting images of synaptophysin relative to actin expression. *Lower panel:* the expression of hippocampal synaptophysin protein relative to actin. (**D**) *Upper panel*: representative immunoblotting images of PSD95 relative to actin expression. *Lower panel:* the expression of hippocampal PSD95 protein relative to actin. NDS: rats with sham operation; NDO: rats with orchiectomy; NDOT: rats with orchiectomy receiving testosterone replacement; NDON: rats with orchiectomy receiving NAC treatment; NDOI: rats with orchiectomy receiving inulin treatment; NDOC: rats with orchiectomy receiving the combined therapy (the combination of NAC and inulin) (N=6 of each group). *p<0.05 in comparison with the NDS, †p<0.05 in comparison with the NDS.

To determine the effects of testosterone deprivation on hippocampal plasticity, dendritic spine density was measured using Golgi staining (Figure 6A and B). The results demonstrated that ORX rats had a significantly decreased dendritic spine density in the CA1 hippocampus, which was attenuated by all treatments (Figure 6B). We further investigated the expression of synaptic proteins by Western blotting, the proteins including synaptophysin and PSD95 (Figure 6C and D). The results showed that ORX rats had significantly decreased levels of both synaptophysin and PSD95 expression, in which were improved by all treatments (Figure 6C and D).

**Figure 7:**
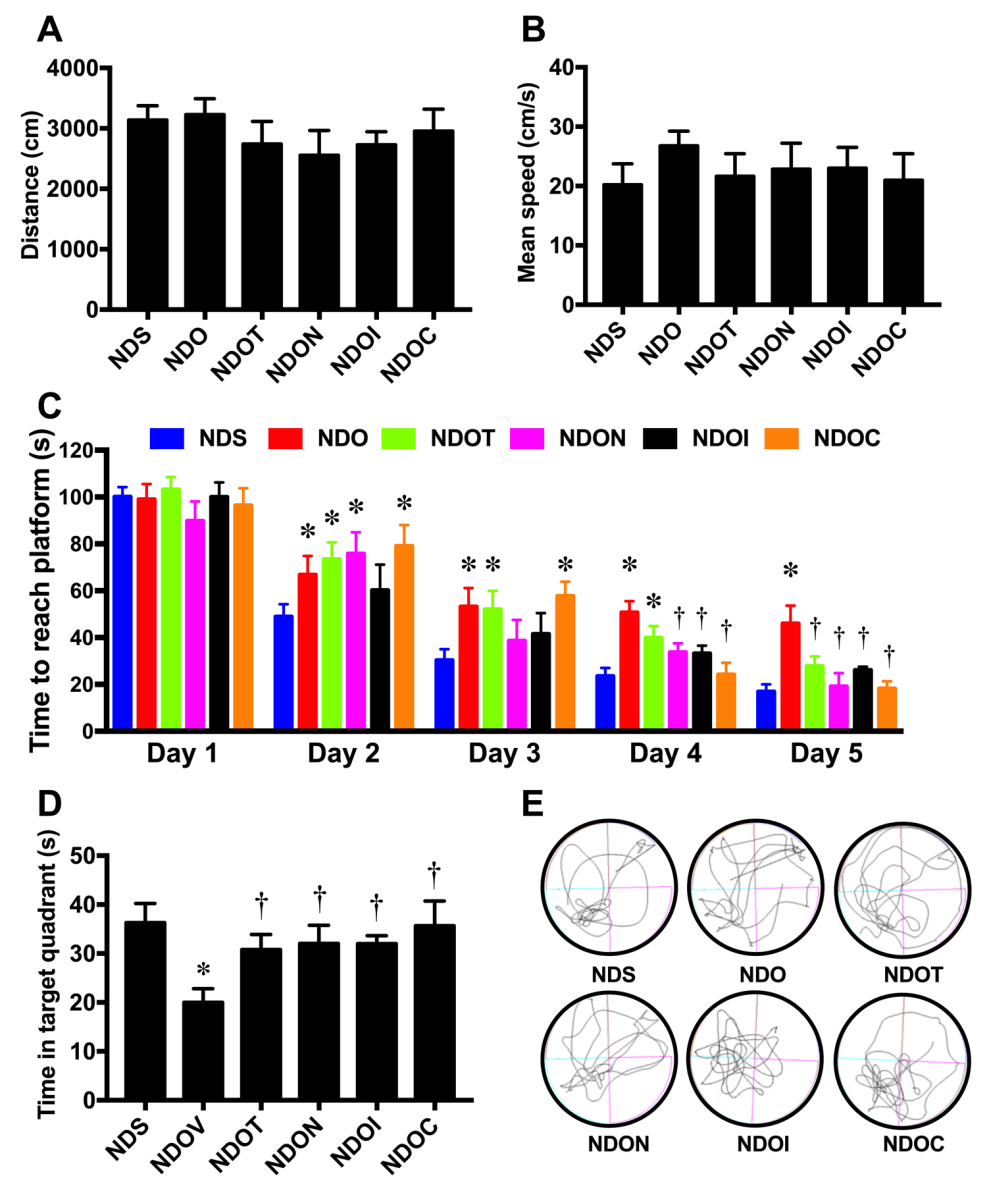
**The effects of NAC, inulin and combined therapy on cognitive function in rats with testosterone deprivation.** (**A**) Locomotor function as indicated by distance during the open-field test. (**B**) Mean speed during acquisition test of Morris Water Maze test. (**C**) Time to reach the platform during acquisition test of Morris Water Maze test. (**D**) Mean time spent in target quadrant during probe test. (**E**) The representative tracing during the probe test (left-lower quadrant is target quadrant). NDS: rats with sham operation; NDO: rats with orchiectomy; NDOT: rats with orchiectomy receiving testosterone replacement; NDON: rats with orchiectomy receiving NAC treatment; NDOI: rats with orchiectomy receiving inulin treatment; NDOC: rats with orchiectomy receiving the combined therapy (the combination of NAC and inulin) (N=6 of each group) *p<0.05 in comparison with the NDS, †p<0.05 in comparison with the NDS.

To determine the effects of testosterone deprivation on cognitive function, the open-field test and the MWM test were performed. We found that locomotor activity was no different between all groups as indicated by no change in distance during open-field tests (Figure 7A). The speed test during MWM test was not significantly different between the groups (Figure 7B). Interestingly, ORX rats demonstrated a longer time taken to reach the platform in the acquisition test (Figure 7C) and a shorter time spent in the target quadrant during the probe test (Figure 7D and E). Testosterone replacement, NAC, inulin, and the combined therapy equally improved cognitive function as indicated by the decreased time taken to reach the platform in the acquisition test and the increased time spent in the target quadrant during the probe test in ORX rats (Figure 7C-E). All of these findings demonstrated that testosterone deprivation caused hippocampal dysplasticity and cognitive decline. Testosterone replacement, NAC, inulin, and the combined therapy attenuated hippocampal dysplasticity, leading to the improvement of cognition in testosterone-deprived condition.

## DISCUSSION

The major findings of this study are as follows: (1) Testosterone deprivation induced gut dysbiosis, BBB dysfunction, increased hippocampal oxidative stress, increased hippocampal inflammation, increased microglial and astrocytic hyperactivity, increased hippocampal apoptosis, and hippocampal dysplasticity, resulting to cognitive decline. (2) Testosterone replacement, NAC, inulin, and the two as a combined therapy equally improved cognitive decline through attenuating gut dysbiosis, improved blood brain barrier function, decreased hippocampal oxidative stress and inflammation, ameliorated glial hyperactivity, decreased hippocampal apoptosis, and restored hippocampal plasticity.

Previous studies have shown that several conditions including dietary changes [11,36,37], stress [38], and antibiotic treatment [39], caused alteration of gut microbiota. These changes were found to be associated with an increased risk of cardiovascular diseases (CVD), T2DM [37], and cognitive dysfunction [11]. Previous study demonstrated that the presence of gut microbes is critical in maintaining a normal estrous cycle in female mice, testosterone levels in male mice, and reproductive function in both sexes [40]. In addition, a previous study in germ-free male mice demonstrated lower systemic testosterone levels compared with conventionally-raised counterparts [40]. Administration of the probiotic *Lactobacillus reuteri* increased circulating testosterone and effectively prevented age-associated testicular atrophy in mice [41]. Similarly, this study showed that testosterone deprivation developed gut dysbiosis by increasing the F/B ratio and the level of *Enterobacteriaceae*. All of these findings emphasize the interaction between testosterone and the gut microbiota. However, it is not clear how testosterone regulates gut microbiota composition. The underlying mechanism of testosterone mediating the level and composition of gut microbiota needs further investigation.

Testosterone deprivation led to develop not only gut dysbiosis, but also increased oxidative stress [42,43]. Our previous study also demonstrated an increase in *Enterobacteriaceae*, which is source of lipopolysaccharide (LPS) [9], led to endotoxemia and low-grade systemic inflammation [11,36]. Both systemic inflammation and oxidative stress affected the blood brain barrier (BBB) function by decreasing tight junction proteins such as claudin-5 and ZO-1 and increasing BBB permeability in testosterone-depleted male mice [44]. The BBB breakdown allowed the brain to be exposed to various cytokines including LPS, interleukin 1 beta (IL-1β), interleukin 6 (IL-6), and tumor necrosis factor alpha (TNFα) [45]. These changes led to further brain oxidative stress [28,29], brain inflammation, and microglial activation [11,29,30], resulting in cognitive decline, as shown in the present study. Testosterone replacement, NAC, inulin, or the combined therapy in ORX rats equally ameliorated cognitive decline, possibly through anti-oxidative and anti-inflammatory effects of these therapies. It has been shown that testosterone and NAC [46,47] had anti-oxidative effects. A prebiotic supplement decreased oxidative status in juvenile White Sea bream [48] and suppressed pro-inflammatory cytokines including IFNγ and IL-1β [49]. Taken together, our findings suggest that BBB dysfunction, brain oxidative stress, and brain inflammation develop as a result of testosterone deprivation, but these deleterious effects can be attenuated by testosterone replacement, NAC, inulin, and the combined therapy.

Growing evidence has demonstrated the crucial roles of microglia and astrocytes on cognitive dysfunction in neurodegenerative disorders [23–25,50]. Recent studies also illustrated the communication links between microglial function and host microbiota [26,27]. Temporary abolishment or limited complexity of host microbiota led to severe changes in microglia function, changes which were restored by recolonization with a complex microbiota [27]. It has been shown that microglia and astrocytes have biphasic roles. One being an inflammatory phenotype (M1 or A1), which was characterized by an amoeboid-like shape [51,52]. The other is an anti-inflammatory phenotype (M2 or A2), characterized by a ramified-like shape [52,53]. A previous study showed negative microglial morphological changes in high fat diet-fed rats, which were improved with prebiotic, probiotic and synbiotics treatment [11]. Recently, our previous study reported that both microglia and astrocytes also had an amoeboid-like shape in castrated male rats similar to this study [29]. All of these findings suggest that testosterone deprivation induced gut dysbiosis and microglial/astrocytic hyperactivity.

In addition to those previous findings, the present study demonstrates that testosterone replacement, or treatment with NAC, inulin, or the combined therapy preserves microglial and astrocytic morphology in testosterone-deprived condition. A previous study similarly demonstrated that testosterone decreases reactive levels of both astrocytes and microglia after brain injury in male rats [54]. Administration of NAC decreased microglial activation and inflammation both in *in* *vitro* and *in* *vivo* situations [32–35]. Surprisingly, we found that the mean fluorescent intensity (MFI) remain increased after testosterone treatment in ORX rats. It is possible that the castration caused brain injury and led to the persistent hyperactivation in microglia and astrocytes via increased androgen receptors. To support this possibility, a previous study reported that microglia and astrocytes increased estrogen and androgen receptors after brain injury [55]. In addition, testosterone replacement for 4 weeks may not be enough time to reduce both microglial and astrocytic hyperactivation in ORX rats. Taken together, these findings demonstrated that NAC, inulin, and the combined therapy effectively attenuated microglial and astrocytic hyperactivity in castrated male rats.

Moreover, our previous studies have shown that testosterone deprivation increased apoptosis, as measured by increasing levels of apoptotic proteins [11]. The present study and other [29] also demonstrated an increase in hippocampal TUNEL positive cells of castrated male rats, resulting to cognitive decline. The underlying mechanisms of neuronal apoptosis in castrated rats may be due to increased oxidative stress and microglial hyperactivation, leading to releases several pro-inflammatory cytokines and brain inflammation [29]. In addition to those previous findings, we found that testosterone replacement, NAC, inulin, or the combined therapy equally decreased hippocampal apoptosis, and improved hippocampal dysplasticity, leading to improve cognition.

It has been shown that a NAC supplement has beneficial effects on cognition by decreasing oxidative stress [47] [56], suppressing inflammation [56], attenuating mitochondrial dysfunction, improving the cholinergic system [47,57] and attenuating energy metabolism imbalance in the brain [57] in various models. Several studies also reported the beneficial effects of inulin on cognitive function in both animal and humans [20–22]. A previous study reported that 10% inulin improved cognitive performance in the light extinction test and the wellbeing of male rats using the functional observational battery assessment tool (FOB) [20]. In addition, a study by Smith et al. demonstrated that consumption of inulin was associated with greater accuracy in a recognition memory task and improved recall performance in healthy subjects [21]. Taken together, these findings suggest that testosterone replacement, NAC, inulin, and the combined therapy equally ameliorated the deleterious effects of testosterone deprivation in the brain leading to the improvement of cognitive decline in castrated male rats. However, the synergistic effects of NAC and inulin in ORX rats were not observed in all parameters. The possible explanation is that the castrated condition and time course of castration in this study may induce only mild pathological condition in the brain, therefore the monotherapy by NAC and inulin can completely mitigate those deleterious effects.

In conclusion, this study demonstrated that testosterone deprivation induced gut dysbiosis, BBB dysfunction, increased hippocampal oxidative stress, increased hippocampal inflammation, increased microglial and astrocytic hyperactivity, increased hippocampal apoptosis, and caused hippocampal dysplasticity, leading to cognitive dysfunction. Testosterone replacement, NAC, inulin, and the combined therapy had equal impact on the improvement in cognition through attenuating the deleterious effects following testosterone-deprived condition. However, no synergistic effects of inulin and NAC were observed in these conditions.

The present study demonstrated that testosterone deprivation not only affected cognitive function, but also altered gut microbiota and increased microglial hyperactivity. The dysbiosis of gut microbiota [58] and cognitive decline were also reported in aging men [59], who developed testosterone deprivation. In addition, since gut microbiota has been shown to regulate the microglia function through the gut-brain axis [27], these findings suggest that gut microbiota and microglia could be therapeutic targets for interventions of cognitive decline under the testosterone-deprived condition. Furthermore, under testosterone-deprived condition, the present study demonstrated that NAC, inulin, a combined therapy (NAC+inulin) and testosterone replacement effectively improved the cognitive function. These findings suggest that prebiotics, antioxidant and the combined prebiotics and antioxidant therapy could provide the beneficial effect on the brain similar to the use of testosterone replacement therapy in testosterone-deprived people.

## MATERIALS AND METHODS

### Animals and diet

**Figure 8:**
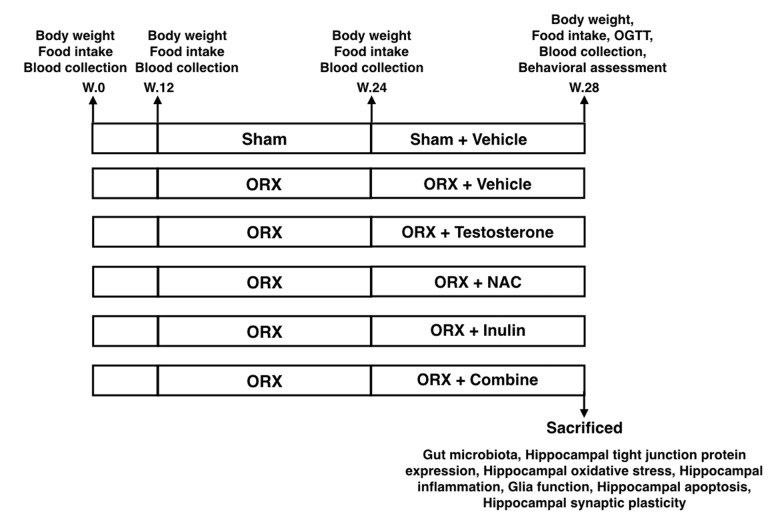
**The experimental protocol of the study OGTT**: Oral glucose tolerance test; ORX: bilateral orchiectomy, Combine: the combination of NAC and inulin.

All animal studies were approved by the Institutional Animal Care and Use Committee (IACUC) of the Faculty of Medicine, Chiang Mai University (Permit Number: 9/2561) and conformed to the Guide for the Care and Use of Laboratory Animals published by the US National Institutes of Health (NIH guide, 8th edition, 2011). Thirty-six male Wistar rats (weight 180 – 200 g) were obtained from Nomura Siam International, Thailand. Animals were housed in a temperature-controlled environment with a 12:12 hour light-dark cycle. After acclimatization for a week, rats were fed on a normal diet (ND; 19.7% E fat) for 34 weeks. All rats had access to reverse osmosis drinking water *ad libitum*. Food intake was recorded daily and body weight was recorded weekly. At week 13, rats were randomly assigned to 2 groups to receive either a sham operation (n=6) or a bilateral orchiectomy (ORX, n=30). Twelve weeks after the orchiectomy, all rats were further subdivided into 6 subgroups (n=6/subgroup) and assigned as follows: ORX rats treated with the vehicle (castor oil), ORX rats treated with testosterone (2 mg/kg of testosterone enanthate dissolved in castor oil, Bayer Schering, Berlin, Germany), ORX rats treated with NAC (oral feeding, 100 mg/kg/day, Sigma, St. Louis, USA), ORX rats treated with inulin (mix with diet, 10% w/w, Orafti® inulin, BENEO GmbH, Germany), and ORX rats treated with the combination of NAC and inulin. Sham-operated rats were treated with the vehicle (castor oil). The treatments in all subgroups were given for 4 weeks. At the end of the 4 weeks, the cognitive function and the oral glucose tolerance test (OGTT) of each rat was investigated. Then rats were deeply anesthetized with isoflurane and terminated by decapitation. The brain and colon content of each rat were quickly removed. The gut microbiota, hippocampal plasticity, tight junction protein expression, hippocampal ROS production, brain inflammation, microglial / astrocyte morphology, and hippocampal apoptosis were determined. The experimental protocol for the study is summarized in Figure 8.

### Orchiectomy

Animals were anesthetized and maintained using 2% isoflurane. After the scrotal area was shaved, an orchiectomy was performed using the scrotal approach technique. Absorbable sutures were used to ligate the blood vessels and vas deferens. Both the testis and the epididymal fat pad were removed. The rats were carefully monitored to prevent the complications. An analgesic drug and antibiotics were injected subcutaneously for three days post-operatively [28,29].

### Blood collection and the determination of metabolic parameters

The rats were fasted for five hours before they were anesthetized using isoflurane. Whole blood was then collected from the tail vein and centrifuged at 6000 rpm for ten minutes. Plasma was collected for biochemical analysis including plasma glucose, insulin, total cholesterol, HDL, triglyceride, and testosterone levels [28,29]. Plasma glucose, cholesterol, triglyceride and HDL levels were measured using a colorimetric assay kit (Biotech, Bangkok, Thailand and Biovision, California, USA). Plasma insulin levels were also measured using the Sandwich ELISA kit (LINCO Research, MO, USA). The Homeostasis Model Assessment (HOMA) was used for assessing peripheral insulin resistance as described in previous study [60]. The OGTT was performed as described in our previous studies [11,29]. Briefly, blood samples were collected from the overnight fasting tail vein rats at 0, 15, 30, 60, 90 and 120 minutes after glucose feeding (2 g/kg). Areas under the curve (AUC) were calculated to evaluate glucose tolerance.

### Determination of gut microbiota

Bacterial genomic DNA samples were extracted from rat fecal pellets using a commercial genomic DNA isolation kit (QIAGEN, Germany). Briefly, the fecal pellet (0.25 g) was homogenized in QIAGEN ASL lysis buffer using a Mini-Beadbeater (BioSpec Products, Bartlesville, OK). The manufacturer’s instructions were then followed to extract the bacterial genomic DNA from the rat fecal pellets. Extracted bacterial genomic DNA was diluted 1:10 and 0.04 mL was used as the template for the SYBR-Green-based (SensiFAST SYBR Lo-ROX kit, Bioline, Taunton, MA) real-time polymerase chain reaction using primers previously described [11,36,61]. The bacterial microbiota population fractions (*Firmicutes*/*Bacteroidetes* ratio and *Enterobacteriaceae*) were calculated using quantitative polymerase chain reaction data (qPCR) as described previously [11,36]. The percentage of each bacterial phylum was determined by dividing by *Eubacteria* level as previously described [11,36,61].

### Determination of hippocampal ROS levels

The methods have been described previously [11,62]. Briefly, the hippocampus was isolated and homogenized in solution buffer containing protease inhibiter. Then, protein levels were determined by BCA assay as described previously [29], with subsequent measurements of the hippocampal ROS levels using dichloro-hydro-fluorescein diacetate (DCFHDA) fluorescent dye.

### Determination of brain MDA levels

The whole brain was homogenized and mixed with 1.1 ml of 10% trichloroacetic acid (TCA) containing butylated hydroxytoluene (BHT) (50 ppm), heated at 90 °C for 30 minutes and cooled down to room temperature. After centrifuged at 6,000 rpm for 10 minutes, the supernatant (0.5 ml) was mixed with 0.44 M H_3_PO_4_ (1.5 ml) and 0.6% thiobarbituric acid (TBA) solution (1.0 ml) and after incubating at 90°C for 30 minutes pink-colored products called thiobarbituric acid reactive substances (TBARS) were shown. The solution was filled through a syringe filter and analyzed using the HPLC system [11,29].

### Immunofluorescent labelling for glial morphology and cell apoptosis

The brain tissue was fixed with 4% paraformaldehyde for 24 hours followed by immersion in 30% sucrose in phosphate-buffered saline (PBS) for an additional 48 hours at 4°C for cryoprotection. The tissue was then frozen in isopentane and dry ice and stored at -80°C. Following this the brains were sliced by cryosection (Leica CM1950, Leica Biosystem Nussloch GmbH, Nussloch, Germany) to produce slices 20 μm thick for glial morphology and 10 μm thick for apoptosis. Sections were subjected to labelling by immunofluorescence. To determine cell apoptosis, the brain sections were processed following the commercial protocol for standard terminal deoxynucleotidyl transferase mediated biotinylated UTP nick end labelling (TUNEL) using the TACS 2TdT-Fluor *in situ* apoptosis detection kit (TACS^Ò^, Trevigen, R and D Systems, Minneapolis, MN, USA). For glial morphology, the sections were quenched using 3% peroxide for one hour, blocked with 5% BSA for one hour, and incubated overnight at 4 °C with primary antibodies for ionization of calcium-binding adapter molecule 1 (Iba-1) (ab5076, Abcam, Cambridge, MA) and glial fibrillary acidic protein (GFAP) (ab16997, Abcam, Cambridge, MA) [11,29]. After being washed three times in PBS, sections were incubated with AlexaFluor conjugated secondary antibodies: Iba1-AlexaFluor 488 anti-goat, GFAP-AlexaFluor 647 anti-rabbit, and DAPI (Tocris Bioscience**,** Bristol, UK), for one hour at 25°C then rinsed three times in TBS. Sections were treated with copper sulfate in ammonium acetate buffer to quench endogenous auto-fluorescence of the brain tissue [11].

### Dendritic spine analysis

To examine dendritic spine density, the brains were transferred to Golgi staining solution. Golgi staining was conducted in accordance with the instructions for the FD Rapid Golgistain™ Kits (FD NeuroTechnologies, Baltimore, MD) on unperfused brain tissue [62,63].

### Image analysis

A series of z-stacks images were taken using confocal microscopy (Olympus fluoview FV3000, Tokyo, Japan) and the glial morphology was analyzed a 3D construction using Imaris software 7.0 (Bitplane, Oxford instrument company, AG, Zurich, Switzerland). Three microglial and astrocyte cells per brain slice from the CA1 region of the hippocampus, three brain slices per animal were measured from all six animals in the group. The mean fluorescent intensity was also measured and the complexity of the glial cells were measured by Sholl analysis [11,27]. A series of z-stack images of TUNEL staining were also analyzed using Imaris software. Three fields per brain slice, three brain slices per animal, and six animals per group were measured for the CA1 region of the hippocampus and cortex [29]. To determine dendritic spine density, three tertiary segments, 100–200 μm apart were taken from the soma. Dendrites 20–30 μm in length were randomly selected and dendritic spine density recorded. Three neuronal cells per brain slice, and three brain slices per animal were selected for spine quantitative analysis. The number of spines were counted by a double-blind hand counter [62,63].

### Open-field test

To determine locomotor activity, all animals were tested using an open-field test [29]. Briefly, each animal was placed into the center of the arena and allowed ten minutes for exploration. After the ten minutes of exploration time, the animals were taken out. The distances the animals had moved were measured using SMART 3.0 software (Panlab, Harvard Apparatus, Barcelona, Spain).

### Morris water maze

In this study the protocol of the MWM was modified from that described by Vorhees et al., 2006 [64]. After 30 minutes of habituation, rats were randomly placed in the water at one out of four starting points with their head turned towards the border of the water pool. After the 15 second interval, the animal was placed at the other three starting points. The same protocol was conducted for five consecutive days for the acquisition test. During the probe test, the platform was removed from the water pool. Animals were placed at the starting point and allowed to swim for 90 seconds. The time to reach the platform during the acquisition test and the time in the target quadrant in the probe test were measured by SMART 3.0 software (Panlab, Harvard Apparatus, Barcelona, Spain) [11,29].

### Statistical analysis

Data from each experiment were expressed as mean ± S.E.M. For acquisition tests, the significance was calculated using repeated Two-way ANOVA tests followed by post-hoc Tukey’s analysis. For all multiple comparisons, data were analyzed using a two-way ANOVA followed by post-hoc Tukey’s analysis. P < 0.05 was considered as statistically significant.
